# Differences in Physiological and Perceptual Responses to High Intensity Interval Exercise Between Arm and Leg Cycling

**DOI:** 10.3389/fphys.2021.700294

**Published:** 2021-08-18

**Authors:** Todd A. Astorino, Danielle Emma

**Affiliations:** Department of Kinesiology, California State University-San Marcos, San Marcos, CA, United States

**Keywords:** interval training, blood lactate concentration, oxygen uptake, exercise mode, affective valence

## Abstract

This study compared changes in oxygen uptake (VO_2_), heart rate (HR), blood lactate concentration (BLa), affective valence, and rating of perceived exertion (RPE) between sessions of high intensity interval exercise (HIIE) performed on the arm (ACE) and leg cycle ergometer (LCE). Twenty three active and non-obese men and women (age and BMI=24.7±5.8year and 24.8±3.4kg/m^2^) initially underwent graded exercise testing to determine VO_2_max and peak power output (PPO) on both ergometers. Subsequently on two separate days, they performed 10 1min intervals of ACE or LCE at 75 %PPO separated by 1min of active recovery at 10 %PPO. Gas exchange data, HR, and perceptual responses were obtained continuously and blood samples were acquired pre- and post-exercise to assess the change in BLa. VO_2_max and PPO on the LCE were significantly higher (*p*<0.001) than ACE (37.2±6.3 vs. 26.3±6.6ml/kg/min and 259.0±48.0 vs. 120.0±48.1W). Mean VO_2_ (1.7±0.3 vs. 1.1±0.3L/min, *d*=2.3) and HR (149±14 vs. 131±17 b/min, *d*=2.1) were higher (*p*<0.001) in response to LCE vs. ACE as was BLa (7.6±2.6 vs. 5.3±2.5mM, *d*=2.3), yet there was no difference (*p*=0.12) in peak VO_2_ or HR. Leg cycling elicited higher relative HR compared to ACE (81±5 vs. 75±7 %HRmax, *p*=0.01), although, there was no difference in relative VO_2_ (63±6 vs. 60±8 %VO_2_max, *p*=0.09) between modes. Affective valence was lower during LCE vs. ACE (*p*=0.003), although no differences in enjoyment (*p*=0.68) or RPE (*p*=0.59) were demonstrated. Overall, HIIE performed on the cycle ergometer elicits higher relative heart rate and blood lactate concentration and a more aversive affective valence, making these modes not interchangeable in terms of the acute physiological and perceptual response to interval based exercise.

## Introduction

Various adaptations to moderate intensity continuous exercise (MICE) include increases in cardiorespiratory fitness (VO_2_max, Church et al., 2007) as well as reductions in blood pressure (Costa et al., 2018) and body fat (Slentz et al., 2004). Together, these responses enhance health status as they are associated with greater cardiometabolic health and in turn, reduced rates of morbidity and mortality (Blair et al., 1996; Kodama et al., 2009). Nevertheless, recent data (Centers for Disease Control, 2020) reveal that only 25% of adults achieve the recommended guideline of 150min/week of MICE, with the primary barrier being lack of time (Trost et al., 2002; Reichert et al., 2007).

One promising alternative to MICE is high intensity interval exercise (HIIE), which includes repeated bouts (approximately 1–5min in duration) of vigorous exercise eliciting intensities >85 percent maximal heart rate (%HRmax) separated by periods of recovery (Weston et al., 2014). Although, these sessions typically require a similar duration as a 30min bout of MICE, they are characterized by a lower training volume and greater time spent at near-maximal intensities which is important to optimize the increase in VO_2_max (Midgley and McNaughton, 2006). In active adults (Milanovic et al., 2015) as well as those with chronic disease (Weston et al., 2014), results from meta-analyses exhibit a superior increase in VO_2_max in response to HIIE compared to MICE when performed long-term. In addition, despite the higher intensities characteristic of HIIE vs. MICE, similar (Stork et al., 2017; Olney et al., 2018) and in some cases greater post-exercise enjoyment (Thum et al., 2017; Oliveira et al., 2018) has been reported that substantiates its broad application as an additional option to engage in physical activity for many adults.

Buchheit and Laursen (2013) stated that the adaptive response to training is mediated by the repeated stress of acute sessions of exercise. Moreover, Egan and Zierath (2013) reported that the molecular mechanisms underpinning these changes are likely due to alterations in muscle mitochondrial protein content and enzyme activity. Consequently, examining the acute physiological response to HIIE is important as it may mediate the magnitude of changes in various outcomes when performed long-term. The majority of data concerning responses to acute HIIE was obtained from leg cycling (Olney et al., 2018), body weight exercise (Gurd et al., 2018), or treadmill running (Nuuttila et al., 2020) that mostly involve the lower extremities. However, results from these studies cannot be generalized to the upper extremity due to its lower amount of muscle mass (Sawka, 1989) and oxidative capacity (Gollnick et al., 1972). Moreover, individuals with joint pain may be intolerant of weight-bearing exercise on the treadmill, body weight exercise, or cycling due to repetitive motion of the knee joint, so upper extremity exercise may be an appropriate alternative exercise modality in some populations.

Previous data in men with spinal cord injury (Brurok et al., 2011) and active non-injured men (Zinner et al., 2016) demonstrate significant increases in VO_2_max, cardiac output, and time trial performance when HIIE arm cycling is performed chronically, yet no data have elucidated the acute response to this modality or compared it to leg cycling. Various adults including those with lower extremity injuries or other impairments may prefer upper-body exercise such as arm ergometry, and there are reports (Cook et al., 1997) of leg pain in response to leg cycling, which may reduce its feasibility in the broader population. Overall, examining acute responses to arm cycling interval-based exercise is an important topic considering the documented efficacy of HIIE and in turn, need for fitness professionals to implement exercise that actually encompasses the demands of high-intensity interval training and can be tailored to the preferences of each client.

The aim of this study was to compare physiological and perceptual responses from an identical session of HIIE between leg (LCE) and arm cycling ergometry (ACE). It was hypothesized that arm cycling will exhibit lower peak VO_2_, heart rate (HR), and blood lactate concentration (BLa) vs. leg cycling due to the smaller amount of exercising muscle mass. In addition, we compared responses between men and women due to prior data (Astorino and Sheard, 2019) showing higher BLa and more aversive affective valence in men vs. women completing acute bouts of HIIE. Women have a smaller upper-body muscle mass compared to men, and this discrepancy may lead to discrepant physiological and perceptual responses to HIIE vs. men.

## Materials and Methods

### Participants

Healthy, non-obese men (*n*=14) and women (*n*=9) who perform resistance training, aerobic exercise, surfing, group exercise, or non-competitive sport for more than 150min/week in the last year completed the study. Their physical characteristics are shown in Table 1. Men had higher body mass and cycling-derived VO_2_max and lower body fat (*p*<0.001) vs. women, although, all other outcomes were not different (*p*>0.10). Across all participants, only three had experience in LCE and none had performed ACE. All completed a standard health-history questionnaire and provided written informed consent to participate in the study, whose procedures were approved by the University Institutional Review Board.

**Table 1:** Participant physical characteristics (mean±SD).

Parameter	Mean±SD	Range	Men	Women
Age (year)	24.7±5.8	20–49	26.0±7.0	24.0±2.0
Gender (men/women)	NA	14/9	NA	NA
Mass (kg)	72.4±12.0	57.0–106.0	78.5±13.0	65.7±6.4^*^
Body mass index (kg/m^2^)	24.8±3.4	21.0–30.5	25.6±2.9	23.6±2.3
Body fat (%)	18.6±7.2	5.2–31.2	15.3±5.9	24.1±5.3^*^
PA (h/wk)	5.9±2.2	3.0–10.5	6.5±2.4	5.6±1.7
VO_2_max (ml/kg/min)	37.2±6.3	31.0–53.8	39.4±6.4	33.7±4.8^*^

SD, standard deviation; PA, habitual physical activity; VO_2_max, maximal oxygen uptake on the cycle ergometer.

* *p*<0.05 vs. men.

### Experimental Design

Participants completed four sessions over a 2–4 week period that were held at the same time of day within participants and separated by at least 48h. The first two visits required incremental exercise to volitional exhaustion to assess peak power output (PPO) and VO_2_max; whereas, the final two sessions consisted of identical bouts of HIIE differing in exercise mode, the order of which was randomized across participants using a Latin Squares design. During all bouts, physiological and perceptual responses were obtained. We instructed participants to eat a light meal 2h before all sessions and to be well-rested, hydrated, and to abstain from physical activity for 36h prior to each session.

### Assessment of Body Composition and VO_2_max

Height and body mass were initially determined to calculate body mass index (BMI). In addition, shoulder, arm, and thigh circumference was measured in rotational order following standardized procedures (Heyward and Gibson, 2014). Subsequently, subcutaneous fat was determined at seven sites using a metal caliper (Lange, Santa Cruz, CA) to determine body density and percent body fat following standardized procedures (Jackson and Pollock, 1978; Jackson et al., 1980). Participants then initiated incremental exercise to volitional exhaustion on an electrically-braked arm ergometer (Lode Angio, Groningen, Netherlands) during which power output was increased in a ramp-like manner by 8 (women) or 15 (men) Watt/min after a 5min warm-up at 7Watt. The pedal crank was aligned to the height of the shoulder joint. Volitional exhaustion occurred when pedal cadence was below 50rev/min. Heart rate was determined using telemetry (Polar, Woodbury, NY), and pulmonary gas exchange data (VO_2_, VCO_2_, V_E_, and RER) were obtained every 15s during exercise using a metabolic cart (ParvoMedics True One, Sandy, UT), which was calibrated before testing following manufacturer guidelines. They returned a minimum of 2days later at the same time of day and completed incremental exercise on an electrically-braked cycle ergometer (Velotron RacerMate, Quark, SD) starting with a 2min warm-up at 40, 50, or 60Watt. Power output increased in a ramp-like manner by 20, 25, or 30W/min and exercise ensued until volitional exhaustion, which was confirmed by pedal cadence less than 50rev/min. Peak power output was identified at the work rate coincident with volitional fatigue, and attainment of VO_2_max was confirmed using the following criteria: change in VO_2_<0.15L/min at VO_2_max; HRmax<10 beats/min of 220 – age, and RER>1.10 (Astorino et al., 2008). Prior to exercise, at the end of the warm-up, and every other minute during incremental testing, participants also provided values of RPE and affective valence as described below.

### HIIE Sessions

Upon arrival, participants completed a brief survey confirming that they met all pre-test guidelines for the session. Subsequently, they completed a 4min warm-up at 10 %PPO followed by 10 1min intervals at 75 %PPO, which was determined from the incremental test. This work rate was chosen as pilot testing revealed that higher intensities during arm cycling may induce premature fatigue. Recovery between intervals lasted for 1min and was performed at 10 %PPO. During the entire session, gas exchange data and HR were obtained every 15s. Values for oxygen uptake and HR for each interval and subsequent recovery were calculated as the average of the four data points, and session VO_2_ and HR were identified as the average value acquired from the entire session (80 data points) excluding the warm-up. Peak HR and VO_2_ (expressed as a percentage of maximum) were calculated as the quotient of the highest mean value from any 1min interval and mode-specific VO_2_/HRmax. In addition, mean VO_2_ and HR values were calculated across all 10 intervals and all 10 recovery periods to represent the cardiorespiratory stress of each phase of the session. Pedal cadence was monitored during the initial HIIE session and maintained during the subsequent session within 5rev/min.

### Assessment of Perceptual Responses and Blood Lactate Concentration

Before all sessions with the participants seated in a chair, participants were read specific instructions according to what each measure represented. They were asked to respond to each scale in terms of their perception at that moment, and their score was repeated to them by the Investigators to ensure that it was accurate. The meaning of the Borg 6-20 RPE scale (Borg, 1982) was communicated by instructing participants to report their exertion based on their level of fatigue, breathing, and HR. The RPE scale is a valid and reliable measure of physical exertion during exercise (Borg, 1982). To describe affective valence (Hardy and Rejeski, 1989), we read the participants the following text: *While participating in exercise, it is common to experience changes in mood. Some individuals find exercise pleasurable; whereas, others find it to be unpleasant. Additionally, feeling may fluctuate across time. That is, one might feel good and bad a number of times during exercise*. This scale is established as a reliable and valid measure of affective state during exercise (Hardy and Rejeski, 1989). These measures were recorded pre-exercise, at the end of the warm-up, at the end of interval 2, 4, 6, and 8, and 30s into intervals 5 and 10. Affective valence (determined using the 11-point Feeling Scale, rating from +5 very good to −5 very bad including 0) was recorded immediately after RPE. About 5min post-exercise, participants were asked to rate the enjoyment of each session using the 18-item Physical Activity Enjoyment Scale (PACES; Kendzierski and DeCarlo, 1991), which is widely used in similar studies studying how acute exercise modifies enjoyment recorded post-exercise (Jung et al., 2014; Thum et al., 2017). After their final HIIE session, participants were asked which modality they would prefer to complete long-term.

Prior to exercise after a 5min seated rest, a 0.7μl blood sample was taken from a fingertip using a lancet (Owen Mumford Inc., Marietta, GA) and portable monitor (Lactate Plus, Sports Research Group, New Rochelle, NY) to assess BLa. The fingertip was cleaned with a damp towel, dried, and then the first drop of blood was wiped away. This measure was repeated 3min post-exercise following identical procedures.

### Monitoring of Dietary Intake

To minimize potential effects of dietary changes on our measures, participants completed a 36h food diary prior to their first HIIE session, which was returned to them to be replicated before the final session.

### Data Analyses

Data are reported as means and SD and were analyzed using SPSS Version 24 (Armonk, NY). We determined the normality of data distributions using the Shapiro-Wilks test. To identify differences in our outcome measures between modalities, two-way repeated measures ANOVA was used, with two levels for modality, and two (BLa), seven (RPE and affective valence), or 22 levels (VO_2_ and HR) for time. Sex was also used as a between-subjects variable in our analyses and results are presented when a significant interaction was shown for that outcome. Paired *t*-test was used to assess differences in enjoyment, energy expenditure, and mean or peak and maximal variables between arm and leg cycling. If a significant F ratio was obtained, Tukey’s *post hoc* test was used to identify differences between means. The Greenhouse-Geisser correction was used if the sphericity assumption was violated. Cohen’s d was used as a measure of effect size, with a small, medium, and large effect equal to 0.2, 0.5, and 0.8, respectively (Cohen, 1988). G Power (Faul et al., 2007) was used to confirm that a sample size of 9 per condition is adequate to detect a change in VO_2_ equal to 0.20L/min across modalities and PACES equal to 10units between men and women. Statistical significance was set at *p*<0.05.

## Results

### Maximal Exercise Responses for LCE and ACE

As expected, VO_2_max (*d*=2.8) and PPO (*d*=6.9) were higher (*p*<0.001) in response to LCE compared to ACE, as was maximal RER (*d*=0.8) and HR (*d*=0.9), although, there was no difference in exercise duration (*p*=0.13, *d*=0.46), RPE (*p*=0.10, *d*=0.51), or affective valence (*p*=0.99, *d*=0.01) recorded at end-exercise. These data are demonstrated in Table 2. VO_2_max derived from ACE was 71.0±12.5% of the value recorded from LCE. VO_2_max obtained from LCE was significantly different between men and women (*d*=0.7), although no difference was shown in response to ACE (27.7±6.8 vs. 24.1±5.8ml/kg/min, *p*=0.21, *d*=0.4).

**Table 2:** Comparison of data from VO_2_max testing between leg and arm cycling ergometry (mean±SD).

Parameter	LCE	ACE	95 %CI	*p* value
VO_2_max (ml/kg/min)	37.2±6.3	26.3±6.6	8–13	<0.001
VO_2_max (L/min)	2.6±0.6	1.9±0.4	0.6–1.0	<0.001
PPO (W)	259.0±48.0	120.0±28.1	127–151	<0.001
HRmax (b/min)	185.4±11.9	176.9±19.2	3–14	0.006
RERmax	1.3±0.1	1.2±0.1	0.01–0.10	0.014
V_E_max (L/min)	112.9±24.3	83.6±21.3	21–38	<0.001
Duration (min)	8.5±1.2	9.1±1.6	NR	0.13
RPE (AU)	17.6±1.5	17.2±1.6	NR	0.10
Affect (AU)	−0.2±2.5	−0.1±2.6	NR	0.99

LCE, leg cycling ergometry; ACE, arm cycling ergometry; 95%CI, 95% CI of the difference between modes; SD, standard deviation; VO_2_max, maximal oxygen uptake; PPO, peak power output; HR, heart rate; RER, respiratory exchange ratio; V_E_, ventilation; RPE, rating of perceived exertion; and NA, not reported.

### Change in Oxygen Uptake and Heart Rate During LCE and ACE

**Figure 1:**
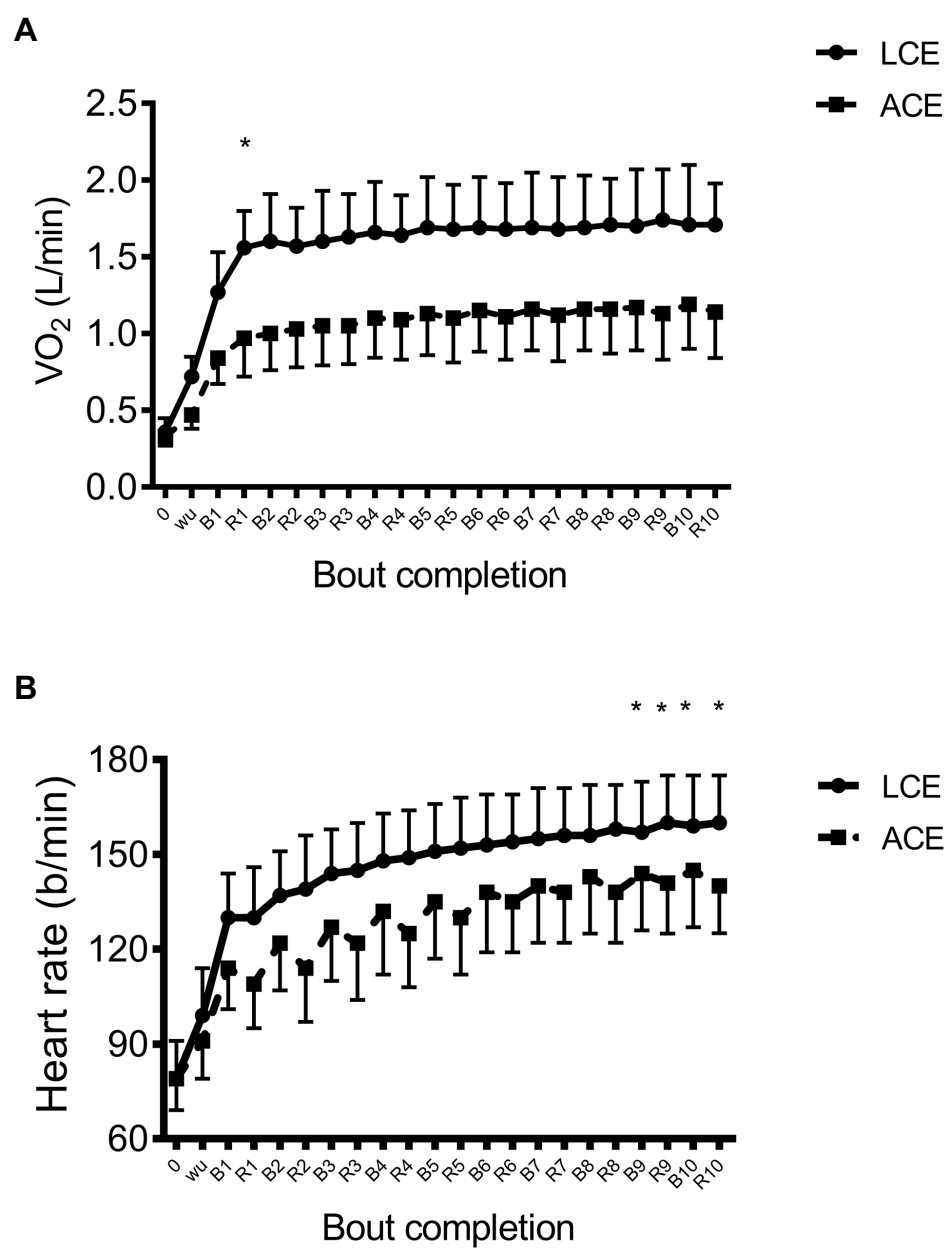
**(A)** VO_2_ and **(B)** heart rate (HR) in response to high intensity interval exercise on the arm ergometer and cycle ergometer; ^*^p<0.05 between modalities.

Figure 1A exhibits the VO_2_ and HR response throughout both sessions of HIIE. Data showed that VO_2_ increased (*p*<0.001) 5-fold from rest during LCE and 3-fold during ACE. There was also a significant effect of modality (*p*<0.001) and significant time X mode interaction (*p*<0.001). Oxygen uptake was consistently 50–60% higher during cycling vs. arm ergometry. Compared to bout 1 and recovery 1, *post hoc* analyses showed higher VO_2_ at bout 2 for both modes (*d*=2.9 and 1.9), which was lower than bout 5 (*d*=1.0 and 1.3), after which VO_2_ was maintained during the subsequent five efforts. Within each modality, there was no difference in VO_2_ between the bout and the corresponding recovery period with exception of bout 1 and recovery 1 for LCE (1.27±0.26 vs. 1.56±0.24L/min, *d*=2.8) and ACE (0.84±0.17 vs. 0.97±0.25L/min, *d*=1.3).

Figure 1B demonstrates the change in HR during exercise and recovery. HR increased substantially (*p*<0.001) from the warm-up to bout 10 during LCE (99±15 vs. 160±15b/min, *d*=1.4) and ACE (91±12 vs. 145±18b/min, *d*=1.2). There was a significant effect of mode (*p*=0.009) yet no time X mode interaction was evident (*p*=0.49). Similar to VO_2_, HR continued to increase during HIIE, although *post hoc* analyses showed that only values in the last two bouts and recovery were significantly higher than bout 1 (*d*=0.68 and *d*=0.70).

### Mean and Peak VO_2_ and HR Response to LCE and ACE

Absolute mean VO_2_ was higher (*p*<0.001, *d*=2.3) in response to LCE compared to ACE (1.7±0.3 vs. 1.1±0.3L/min) as was energy expenditure (161±27 vs. 105±24kcal, *p*<0.001, *d*=2.4); however, there was no difference in relative VO_2_ (63±6 vs. 60±8 %VO_2_max, *p*=0.09, *d*=0.51) or peak VO_2_ (70.5±5.8 vs. 67.2±8.4 %VO_2_max, *p*=0.12, *d*=0.48) between modes. There was no difference (*p*=0.12, *d*=0.46) in peak HR between modes (88±6 vs. 85±8 %HRmax), although, mean HR was higher (*p*<0.001, *d*=2.1) in response to LCE vs. ACE (149±14 vs. 131±17b/min and 81±5 vs. 75±7 %HRmax, *p*=0.01, *d*=1.0). Similar results were shown for peak V_E_, which was higher (*p*<0.001, d=1.5) in response to LCE compared to ACE (67.6±17.4 vs. 48.0±11.2L/min). Results showed a significantly higher BLa (*p*<0.001, *d*=1.3) in response to HIIE on the LCE compared to ACE (7.6±2.6 vs. 5.3±2.5mM).

Data showed no difference in the mean VO_2_ response from intervals 1–10 vs. that attained in recovery after each interval for LCE (1.62±0.32 vs. 1.66±0.38L/min, *p*=0.29, *d*=0.3) and ACE (1.11±0.27 vs. 1.09±0.29L/min, *p*=0.60, *d*=0.1). Nevertheless, paired *t*-test showed significantly higher (*p*=0.001, *d*=1.3) mean HR in recovery from LCE (155±14b/min) compared to that elicited in each interval (151±14b/min). For ACE, the 10 intervals elicited higher HR (*p*=0.001) vs. subsequent recovery (138±17 vs. 131±18±b/min, *d*=1.2).

### Change in Perceptual Responses to LCE and ACE

**Figure 2:**
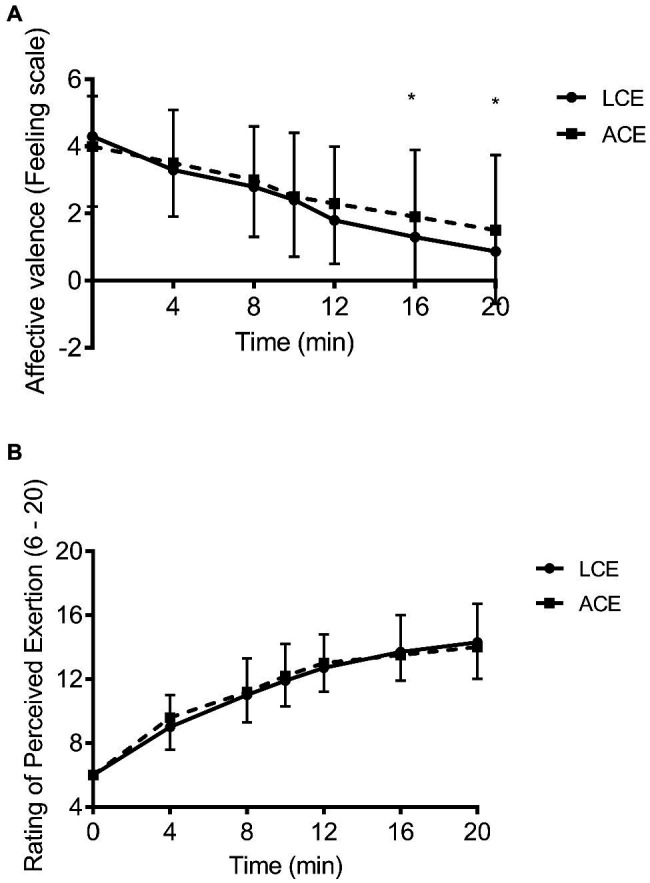
**(A)** affective valence and **(B)** rating of perceived exertion in response to high intensity interval exercise on the arm ergometer and cycle ergometer; ^*^p<0.05 between modalities.

The change in affective valence and RPE is shown in Figures 2A,B. Affective valence declined during exercise (*p*<0.001) and there was a significant time X mode interaction (*p*=0.003) although no effect of mode (*p*=0.49). *Post hoc* analyses revealed more positive affective valence after bout 8 (1.9±1.9 vs. 1.3±2.6, *d*=0.95) and during bout 10 (1.5±2.2 vs. 0.9±2.8, *d*=0.99) for ACE vs. LCE. No time X sex (*p*=0.08), mode X sex (*p*=0.65), or time X mode X sex interaction (*p*=0.60) was revealed for affective valence. As expected, RPE significantly increased during HIIE (*p*<0.001), although, there was no time X mode interaction (*p*=0.59) or main effect of mode (*p*=0.76). *Post hoc* analyses showed that all RPE values were significantly different from each other during HIIE irrespective of modality (*d*=0.4–1.4). Results showed a time X sex interaction (*p*=0.01) as RPE was higher in women vs. men throughout HIIE for both modalities with exception of bout 8 and end-exercise. Compared to men, RPE in women completing LCE was higher after bout 2 (10±2 vs. 8±2, *d*=1.4), 4 (13±1 vs. 10±2, *d*=1.7), 5 (13±1 vs. 11±2, *d*=1.4), and 6 (14±1 vs. 12±2, *d*=1.3), and similar findings were shown for ACE (10±2 vs. 9±2, 12±1 vs. 11±2, 13±2 vs. 12±2, and 14±2 vs. 12±2, *d*=0.8–1.2). Data showed no difference (*p*=0.68, *d*=0.12) in PACES between modes (93.6±18.1 vs. 95.6±18.8 for LCE and ACE, respectively) and 54% of individuals identified LCE as their preferred exercise mode, while 46% preferred ACE.

### Differences in HR, BLa, and PACES Between Men and Women

**Figure 3:**
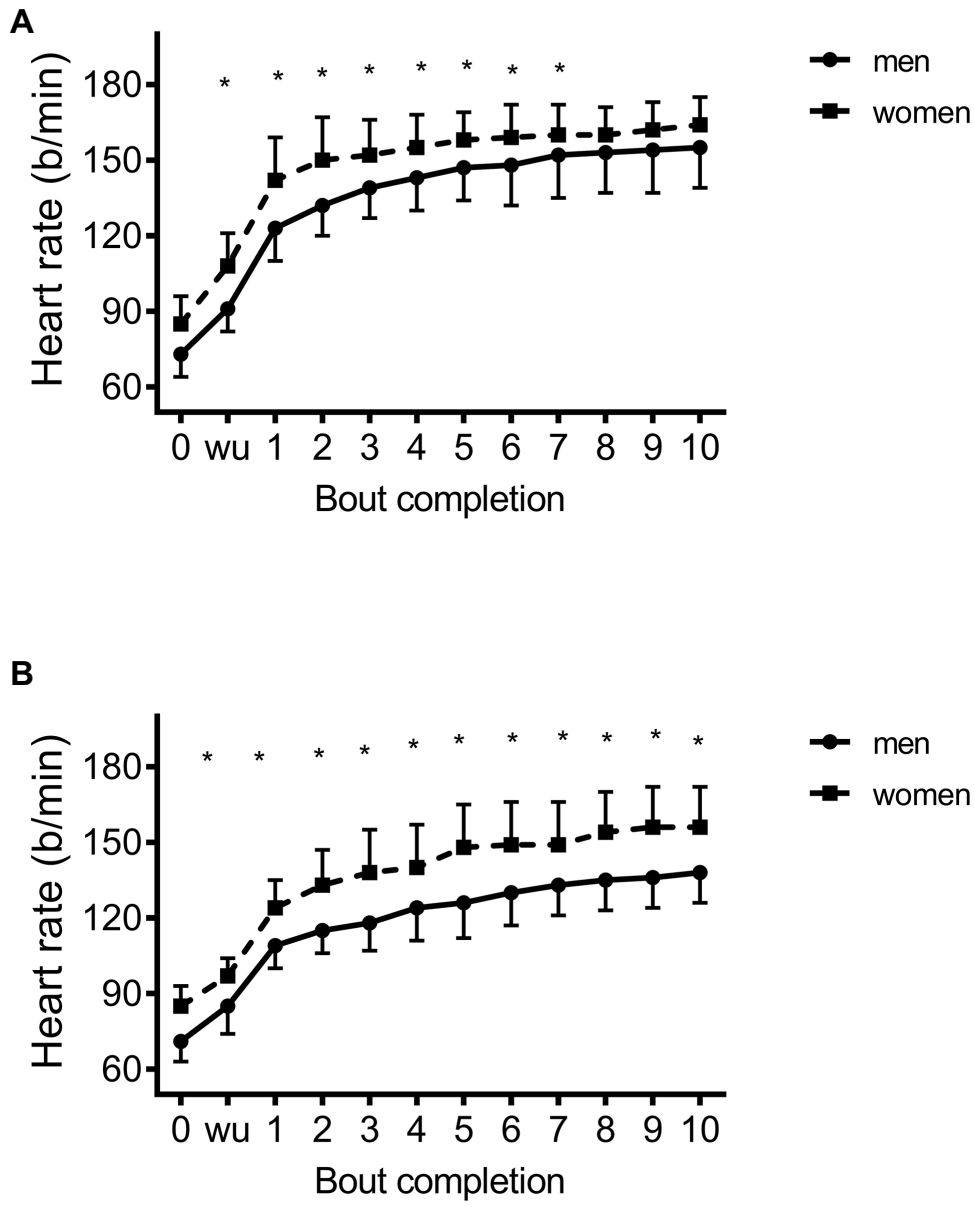
Heart rate response between men and women during high intensity interval exercise on the **(A)** cycle ergometer and **(B)** arm ergometer; ^*^p<0.05 between men and women.

Results showed a significant time X mode X sex interaction (*p*<0.001) as women showed higher HR throughout HIIE during LCE (8–18b/min higher, *d*=1.3–3.3) and ACE (12–20b/min higher, *d*=2.4–4.0) vs. men (Figures 3A,B). *Post hoc* analyses showed that all values were significantly different between men and women other than those acquired in bouts 7–10 for LCE, and at rest for ACE. However, there was no difference (*p*>0.07–0.25) between men and women for LCE (88±4 vs. 86±7 %HRmax and 79±5 vs. 83±5 %HRmax for peak and session HR) or ACE (87±4 vs. 83±9 %HRmax and 73±8 vs. 77±7 %HRmax). Mean oxygen uptake was also not different (*p*>0.17) between men and women in response to LCE (62±7 vs. 64±4 %VO_2_max) or ACE (62±8 vs. 57±6 %VO_2_max). Similarly, data showed no differences in BLa between men and women in response to LCE (8.2±3.2 vs. 6.7±1.4mM, *p*=0.22) or ACE (5.3±1.5 vs. 5.3±1.6mM, *p*=0.98). No differences were also shown in PACES in response to LCE (89.8±17.8 vs. 99.8±18.0, *p*=0.21) or ACE (94.1±14.8 vs. 97.9±21.0, *p*=0.65).

## Discussion

Prior data reveal the efficacy of cycling-based high intensity interval training using the 10×1 protocol to improve cardiorespiratory fitness (Astorino et al., 2013) in inactive adults. However, interval-based cycling is not feasible for all individuals and an alternative mode, arm cycling ergometry, has been shown to elicit enhanced cardiorespiratory fitness and exercise performance (Zinner et al., 2016), although, acute responses to this modality are poorly understood which casts doubt whether requisite intensities characteristic of HIIE can be induced during ACE. This study compared physiological and perceptual responses to HIIE on the arm and cycle ergometer, and results show that LCE elicits higher relative HR and blood lactate concentration and a more negative affective valence compared to ACE at the same relative intensity, making these modes not interchangeable in terms of their acute physiological and perceptual response.

High intensity interval exercise is typically defined as bouts eliciting peak intensities equal to or above 85 %HRmax (Weston et al., 2014) which both modalities attained (see Results). Due to the small muscle mass activated during ACE and potential for premature fatigue to result, we selected a relatively low work rate equal to 75 %PPO, which was identical to that performed during LCE. Mean HR was higher during LCE compared to ACE, representing a “large effect,” which is supported by prior data from graded exercise showing higher HR for combined leg/arm exercise compared to arm exercise alone (Hoffmann et al., 1996). Our data also show that mean and peak VO_2_ was not different during LCE compared to ACE; however, absolute VO_2_ was significantly higher in response to LCE. The enhanced VO_2_ inherent with LCE leads to higher energy expenditure and potentially a greater caloric deficit if maintained long-term. There are reports that running-based HIIE elicits greater changes in body composition than leg cycling (Wewege et al., 2017), likely due to the greater muscle mass activated. However, these differences are modest and additional work is needed to ascertain if exercise modality exudes a significant effect on magnitude of body fat loss associated with interval training. Midgley and McNaughton (2006) reported that superior increases in cardiorespiratory fitness occur when endurance exercise training is performed at higher intensities relative to VO_2_max. However, more recent data show that divergent intensities of cycling-based HIIE (expressed using %PPO) elicit similar increases in VO_2_max in active and inactive adults (Astorino et al., 2013, 2017; Matsuo et al., 2014). In active men, Zinner et al. (2016) reported a significantly greater increase in VO_2_max in response to sprint interval exercise performed using ACE vs. LCE despite lower work completed. However, the power outputs completed during ACE were higher relative to fat free mass compared to LCE, which may explain this discrepant response. Our results show that peak VO_2_ and HR are not different between modes, suggesting that the peak cardiorespiratory strain during HIIE is similar during exercise having different amounts of exercising muscle mass.

In response to repeated Wingate tests, Hazell et al. (2014) showed that VO_2_ frequently attained its highest values in recovery rather than during each of the four intervals. For example, their results demonstrated that VO_2_ attains 88–99 %VO_2_max during recovery that was markedly higher than relative intensities elicited during each 30s sprint (53–72 %VO_2_max). Our data albeit for a less intense regimen of HIIE reveal similarly high values for VO_2_ in recovery vs. that shown from each interval, which is likely due to the relatively short duration of each interval. This sustained elevation in VO_2_ throughout our entire 20min session of exercise promotes a higher overall energy expenditure that may be important for weight loss, especially considering that only 15min of exercise per day is needed to prevent weight gain (Hill et al., 2003).

Changes in affective valence during exercise may predict long-term adherence (Williams et al., 2008) making this outcome important to measure in studies comparing discrepant exercise protocols or modalities. Our data show more positive affective valence representing a “large effect” during ACE compared to LCE, which is potentially due to the lower BLa accumulation. It has been reported that BLa accumulation during HIIE is significantly and inversely associated with the change in affective valence (Astorino and Vella, 2018). However, our data showed no difference in post-exercise enjoyment or RPE between LCE and ACE as both modalities elicited a peak value representing “hard.” This latter finding occurred despite LCE eliciting a higher relative HR and BLa vs. ACE. However, an intriguing finding is that women reported markedly higher RPE values representing a “large effect” during LCE and ACE compared to men. It is likely that the lower cycling-derived VO_2_max of our female participants as well as their likely smaller upper body muscle mass engaged in ACE would require greater force production per muscle fiber and lead to greater sensory strain, in turn augmenting RPE vs. men. This higher RPE occurred despite women exercising at similar fractions of maximal HR/VO_2_ during HIIE and exhibiting similar BLa and affective valence vs. men (see Results). Although, there are reports that HIIE can be prescribed according to RPE (Ciolac et al., 2015), based on our results, this approach may be inappropriate in studies using interval exercise consisting of ACE or LCE, although, further work is needed to substantiate this in a larger sample of women.

This study has a few limitations. First, data do not apply to older men and women who are inactive or obese or to weight bearing exercise modalities such as running, which has a higher energy expenditure than both LCE and ACE. Second, these findings only apply to the specific intensity selected equal to 75 %PPO, and it is unclear if higher intensities including supramaximal workloads as used in sprint interval exercise would exhibit similar responses. For example, previous data (Wood et al., 2016) acquired in active adults show higher VO_2_ in response to HIIE compared to SIE performed using LCE. Third, the order of VO_2_max testing was not randomized as ACE was always performed first, and it is possible that a small effect of learning may have been experienced during the subsequent bout of LCE. However, this work is strengthened by the large and heterogeneous sample divergent in sex and cardiorespiratory fitness as well as precise determination of work rates based on PPO rather than %HRmax, which may be inappropriate for exercise programming using HIIE. Also, we assessed VO_2_ and HR in recovery between bouts to more thoroughly describe the cardiorespiratory stress of interval-based exercise.

Our results show that HIIE performed on the LCE elicits higher mean HR, blood lactate concentration, energy expenditure, and less positive affective valence vs. ACE, so these modes provide discrepant cardiometabolic and perceptual strain. However, there is no difference in peak VO_2_, HR, exercise enjoyment, or RPE between modalities. If achieving a caloric deficit is the primary goal of exercise programming, it appears that HIIE on the cycle ergometer is the preferred modality since it elicits markedly higher energy expenditure than arm cycling. Also, the sex difference in RPE demonstrates that women perceive greater exertion during these HIIE modalities compared to men, and additional work is needed to elucidate this response.

## Data Availability Statement

The original contributions presented in the study are included in the article/Supplementary Material; further inquiries can be directed to the corresponding author.

## Ethics Statement

The studies involving human participants were reviewed and approved by CSU-San Marcos IRB. The patients/participants provided their written informed consent to participate in this study.

## Author Contributions

TA developed the study, participated in data collection, recruited participants, analyzed the data, and created the final draft of the manuscript. DE partook in participant recruitment, data collection, and reviewed the final draft of the manuscript. All authors contributed to the article and approved the submitted version.

## Conflict of Interest

The authors declare that the research was conducted in the absence of any commercial or financial relationships that could be construed as a potential conflict of interest.

## Publisher’s Note

All claims expressed in this article are solely those of the authors and do not necessarily represent those of their affiliated organizations, or those of the publisher, the editors and the reviewers. Any product that may be evaluated in this article, or claim that may be made by its manufacturer, is not guaranteed or endorsed by the publisher.
